# Roles of Oxidative Stress in Polycystic Ovary Syndrome and Cancers

**DOI:** 10.1155/2016/8589318

**Published:** 2015-12-06

**Authors:** Tao Zuo, Minghui Zhu, Wenming Xu

**Affiliations:** ^1^Joint Laboratory of Reproductive Medicine, SCU-CUHK, West China Second University Hospital, Sichuan University, Chengdu, Sichuan 610041, China; ^2^Department of Biological and Pharmaceutical Engineering, School of Chemical Engineering, Sichuan University, Chengdu, Sichuan 610065, China; ^3^Key Laboratory of Birth Defects and Related Diseases of Women and Children of Ministry of Education, West China Second University Hospital, Sichuan University, Chengdu 610041, China; ^4^Reproductive Medicine Center, The Second Affiliated Hospital of Chengdu University of Traditional Chinese Medicine, Chengdu, Sichuan 610041, China

## Abstract

Oxidative stress (OS) has received extensive attention in the last two decades, because of the discovery that abnormal oxidation status was related to patients with chronic diseases, such as diabetes, cardiovascular, polycystic ovary syndrome (PCOS), cancer, and neurological diseases. OS is considered as a potential inducing factor in the pathogenesis of PCOS, which is one of the most common complex endocrine disorders and a leading cause of female infertility, affecting 4%–12% of women in the world, as OS has close interactions with PCOS characteristics, just as insulin resistance (IR), hyperandrogenemia, and chronic inflammation. It has also been shown that DNA mutations and alterations induced by OS are involved in cancer pathogenesis, tumor cell survival, proliferation, invasion, angiogenesis, and so on. Furthermore, recent studies show that the females with PCOS are reported to have an increasing risk of cancers. As a result, the more serious OS in PCOS is regarded as an important potential incentive for the increasing risk of cancers, and this study aims to analyze the possibility and potential pathogenic mechanism of the above process, providing insightful thoughts and evidences for preventing cancer potentially caused by PCOS in clinic.

## 1. Introduction

Polycystic ovary syndrome (PCOS) is one of the most common endocrine disorders of women at reproductive age and the major cause of anovulatory infertility [1]. It was first described as the change of ovarian morphology by Chereau in 1844 [2], and the diagnostic criteria were established by the European Society for Human Reproduction and Embryology (ESHRE) and American Society for Reproductive Medicine (ASRM) in 2003 based on the extensive studies during the last decades, which is the so-called Rotterdam Consensus Criteria [3]. PCOS is a disease with high heterogeneity, and its clinical features mainly include menstrual disorder, secondary amenorrhea, serum hormone abnormality, hairiness, acne, obesity, and infertility [3].

PCOS has been regarded as a chronic systemic disease instead of the simple local disease, and it is frequently associated with insulin resistance (IR), hyperandrogenemia, chronic inflammation, and oxidative stress (OS), though the pathogenesis mechanism has not been well defined [4–8]. A lot of investigations have revealed that OS level is significantly increased in patients with PCOS compared with the normal, when oxidative status is evaluated by circulating markers, such as malondialdehyde (MDA), superoxide dismutase (SOD), and glutathione peroxidase (GPx) [4]. However, OS level is also observed to be significantly correlated with obesity, insulin resistance, hyperandrogenemia, and chronic inflammation [9–12]. Though OS is considered as a potential inducement of PCOS pathogenesis [4], it is still undetermined whether the abnormal OS levels of patients with PCOS derive from PCOS itself or if they are related to the potential complications.

Besides the above complications, PCOS is probably accompanied with some malignant lesions as well, such as endometrial cancer, breast cancer, and ovarian cancer [13, 14]. Several investigations indicated that PCOS perhaps could increase the risk of developing endometrial cancer, and abnormal hormone level, IR, hyperinsulinemia, and even obesity were suggested as the potential inducements of endometrial cancer pathogenesis in PCOS patients [15–18]. What is more, OS, altered in PCOS, is discovered to play pivotal roles in cancer pathogenesis [19–21]. ROS could cause genetic changes by attacking DNA, leading to DNA damages, such as DNA strand breaks, point mutations, aberrant DNA cross-linking, and DNA-protein cross-linking [22]. As a result, the mutations in protooncogenes and tumor suppressor genes probably hijacked cell proliferation out of control, when the DNA repair mechanism has been disrupted [23, 24]. On the other hand, OS could cause epigenetic changes as well by DNA methylation, silencing tumor suppressor genes [25, 26]. Therefore, OS could be one of the major underlying inducements of the increasing risk of gynecological cancers in PCOS patients.

## 2. Altered Oxidative Stress in Polycystic Ovary Syndrome

Oxidative stress (OS) reflects an imbalance between production and scavenging of reactive oxygen/nitrogen species (ROS/RNS) [27], and excess ROS accumulated in vivo would induce cell [28, 29], protein [30–32], and lipid damage [33]. ROS includes both free radical and non-free radical oxygenated molecules, such as hydrogen peroxide (H_2_O_2_), superoxide (O_2_^∙−^), singlet oxygen (1/2 O_2_), and the hydroxyl radical (^∙^OH). Reactive nitrogen, iron, copper, and sulfur species are also involved in OS [34, 35]. Free radicals are the species possessing unpaired electron in the external orbit and could exist independently [35, 36]. In general, chemical substances used for evaluating oxidative status could be divided into chemical components modified by reactive oxygen, ROS scavenging enzymes or antioxidative chemicals, and transcription factors regulating ROS production. However, it is hard to reflect OS status accurately with the same biomarkers in various diseases, because OS usually plays different roles and triggers different signaling pathways in different diseases, so biomarkers used to evaluate OS in a particular disease are limited and should be always filtrated carefully [32, 37–40].

According to the modified criteria defined at Rotterdam meeting, polycystic ovary syndrome (PCOS) would be determined when two of the following three criteria have been discovered: (1) clinical and/or biochemical evidence of androgen excess after the exclusion of other related disorders; (2) oligoovulation or anovulation; (3) ultrasound appearance of the ovaries: presence of more than 12 follicles in each ovary measuring 29 mm and/or increased ovarian volume (>10 mL) [3]. Though the full pathophysiology of PCOS is still not determined, hyperandrogenemia and insulin resistance (IR) are frequently involved. The hyperandrogenemia that accompanies PCOS may be caused by the abnormal ovaries, adrenal glands, peripheral fat, and hypothalamus-pituitary compartment. Insulin resistance, frequently appearing in PCOS as well, results in a compensatory hyperinsulinemia, which augments luteinizing hormone- (LH-) stimulated androgen production, either via its own receptors or via insulin growth factor (IGF-1) receptors [41]. As a syndrome, PCOS is usually treated based on detailed clinical symptoms, and therapeutic schedules mainly include ovulation induction, downregulating androgen and LH levels, attenuating IR, and operation [41].

OS is also intimately involved in PCOS pathogenesis, since PCOS patients show more serious OS compared with the normal [4] (Table 1). However, results would not be consistent absolutely, when different markers are employed and the same marker is evaluated in different sources and even with different investigation methods [42–44]. In addition, OS is involved in the pathological processes of IR, hyperandrogenemia, and obesity as well, which accompany PCOS frequently but not absolutely [45]. Thus, appropriate markers should be chosen to evaluate the OS levels in PCOS for the particular circumstance. Current employed circulating markers majorly include homocysteine, malondialdehyde (MDA), asymmetric dimethylarginine (AMDA), superoxide dismutase (SOD), glutathione (GSH), and paraoxonase-1 (PON1) [4]. Because of the complicated cross-link of OS and physiological and clinical characteristics of PCOS, the interactions of OS and PCOS would be described below from major nodes linking OS and PCOS.

### 2.1. Oxidative Stress, Obesity, and Polycystic Ovary Syndrome

Obesity, a popular endocrine disease in the world, was firstly divided into visceral obesity and peripheral obesity by Vague in 1956 [46], also called central obesity and lower body obesity. Visceral obesity, the so-called abdominal obesity, in which visceral adipose tissues are mainly accumulated in the abdomen and distributed widely on omentum and mesenterium, around viscera, and in skeletal muscle, could be determined by the increased waist circumference (WC). Compared with visceral obesity, peripheral adipose tissues are mainly accumulated under the peripheral skin, especially in buttocks and legs, and are usually evaluated by body mass index (BMI). About 42% of patients with polycystic ovary syndrome (PCOS) have the complication of obesity [47]. Abdominal adipose tissue is considered to be correlated with metabolic diseases more significantly than subcutaneous adipose tissue [48]. Diagnostic method of abdominal obesity has not been defined yet, but the size and the thickness of visceral fat determined by electronic computer X-ray tomography technology (CT) are often regarded as the golden standard [49]. In addition, WC is a simple and reliable criterion usually applied to evaluate abdominal obesity in clinic. Abdominal obesity is regarded as a common complication of PCOS, and the risk of abdominal obesity in PCOS women ranges from 40% to 80% because of the differences of people and nations [50, 51]. Body mass index (BMI) is used as a popular criterion in clinic to evaluate obesity; however, about 50% of PCOS patients with normal BMI still have abdominal obesity [51]. Therefore, both BMI and WC should be considered when considering the contribution of obesity to PCOS etiology.

Obese patients are expected to have more serious oxidative stress (OS) levels [52], and significant correlations of OS markers with obesity indexes, such as BMI and WC, are discovered [53, 54]. Levels of markers that could reflect the degrees of lipid peroxidation and protein peroxidation, such as oxidized low density lipoprotein (ox-LDL), malondialdehyde (MDA), thiobarbituric reactive substances (TBARS), and advanced oxidation protein products (AOPP), increase significantly in the obese patients compared with the normal, and levels of markers that could reflect the antioxidant ability, such as glutathione peroxidase (GSH-Px) and copper- and zinc-containing superoxide dismutase (CuZn-SOD), decreased significantly [55–57]. As an important pathological and physiological process, OS is associated with a number of chronic diseases, which are the main complications of obesity. What is more, the investigation of Khan et al. [58] reported that systemic OS levels of obese females without smoking history, diabetes, hypertension, dyslipidemia, dysfunctions of liver and kidney, and tumor history were still significantly higher than nonobese females, and GSH concentrations of erythrocytes were significantly lower. In addition, obese patients have more serious oxidative stress as well while PCOS patients are ruled out [9, 59]. Thus, obesity, besides abdominal obesity, is directly associated with OS and contributes to the increased OS levels in PCOS [60].

However, obesity is not the only factor leading to the more serious oxidative status of PCOS, and other factors are considered to have contributions as well. While obese patients are ruled out according to BMI, nonobese women with PCOS still have more serious oxidative stress compared with those without PCOS (Table 1). What is more, when PCOS patients with abdominal obesity are excluded instead of peripheral obesity, the result remains the same [61]. In conclusion, obesity is a one of the impact factors contributing to the increased OS levels in PCOS but not the only one.

### 2.2. Oxidative Stress, Insulin Resistance, and Polycystic Ovary Syndrome

Insulin resistance (IR) is a physiological condition in which a given concentration of insulin produces a less-than-expected biological effect, because cells fail to respond to the normal actions of the hormone insulin, leading to dysfunctions of glucose transfer and utilization [62, 63]. Andres clamp technique is the most accurate method to diagnose IR, but its high cost limits the clinical acceptance; therefore, fasting insulin (FINS) and homeostasis model assessment of insulin resistance (HOMA-IR) are usually employed in clinic [64, 65]. IR is regarded as the core mechanism of polycystic ovary syndrome (PCOS) pathogenesis [3], and the IR rate of PCOS patients ranges from 50% to 70% [66, 67]. In fact, IR markers of women with PCOS, such as HOMA-IR, increase significantly compared with normal women and are usually significantly correlated with oxidative stress (OS) markers [10, 68, 69].

IR encourages OS because hyperglycemia and higher levels of free fatty acid lead to reactive oxygen species (ROS) production [45, 70]. When excess glucose or free fatty acid are absorbed in the cell, a large number of reducing metabolites, just like pyruvic acid and acetyl coenzyme A, will be transferred into mitochondria for oxidization, leading to enhancing the activity of electron transport chain and single electron transfer, finally resulting in increasing ROS production. Furthermore, OS would be caused if reducing enzymes, just like super oxidative dismutase (SOD), peroxidase, and catalase, fails to scavenge the excess ROS in the cell [27, 71]. In the IR model of animals induced by high fructose, OS is observed to be enhanced, with the increased protein carbonyl, nonesterified fatty acid (NEFA) and malondialdehyde (MDA), O_2_^−^, reduced glutathione (GSH), and so on [72–74]. As it is known, IR is frequently accompanied with obesity and exists in about half of the obese [47], so IR is also regarded as one of the core mechanisms by which obesity contributes to OS. In the study of Huber-Buchholz et al., reducing the body weight by 11%, obese women were demonstrated to increase insulin sensitivity by 71% and decrease fasting insulin levels by 33% [75]. However, the correlation of oxidative stress and IR is still significant independent of obesity [10].

Though the full mechanism of OS-induced IR remains unclear, OS has been demonstrated to play crucial roles in IR pathogenesis [70, 76]. In multiple studies, it was reported that exposure to oxidative stress inhibits the metabolic pathways induced by insulin in L6 myotube and 3T3-L1 adipocyte models [77, 78]. According to the investigation of Bloch-Damti and Bashan, insulin-stimulated glucose uptake, glycogen synthesis, and protein synthesis would be inhibited after exposure to 50 *μ*M H_2_O_2_ for 2 hours [70]. Oxygen radical plays an important role in glucose regulation [79]. For example, H_2_O_2_ could regulate the insulin release of *β* cell stimulated by glucose and participate in the regulation of insulin signaling pathway [80]. In general, insulin receptor substrate (IRS) is the key player of IR pathogenesis [81]. With the increased OS, various protein kinases are activated to induce serine/threonine phosphorylation of IRS and inhibit normal tyrosine phosphorylation of IRS, reducing the capacity of IRS to combine with insulin receptor, suppressing IRS to activate the downstream phosphatidyl inositol 3-kinase (PI3K); and finally insulin signal to the effector via insulin receptor (InsR)/IRS/PI3K pathway is interfered with. In addition, serine/threonine phosphorylation of IRS could also induce the degradation of IRS and make IRS become the inhibitor of InsR kinase [82, 83]. Insulin signaling pathways could also be activated by OS mainly through Jun N-terminal kinase/Stress Activated Protein Kinase (JNK/SAPK) signaling pathway and inflammatory signaling pathway (I*κ*B kinase/nuclear factor *κ*B, IKK/NF-*κ*B), leading to IR via post-insulin receptor defect [84–86].

IR in PCOS is alternative for glycometabolism, and the synthesis of sex hormones is enhanced [87, 88]. The mechanism of the alternative IR in PCOS still remains unclear, but post-insulin receptor defect in insulin signaling is regarded as the major pathogenesis mechanism of IR in PCOS [89]. Levels of Ser-phosphorylated IRS-1 of adipose tissue and serum in PCOS women are significantly higher than those in controls, whereas IRS-1 tyrosine phosphorylation levels in PCOS women are lower than in controls [90, 91]. The amount of IRS-1 decreases in adipose tissue and granulosa cells but increases in PCOS theca cells [61, 92]. Levels of activated extracellular signal-regulated kinase 1/2 (ERK1/2) of adipose tissue and serum in PCOS women are observed to be higher than those in controls, but levels of insulin receptor, glucose transporter-4 (GLUT4), and PI3K are lower [61, 90].

Thus, OS is intimately associated with IR and is possible to be the major inducement of IR in PCOS via post-insulin receptor defect. In addition, studies with antioxidants such as vitamin E, *α*-lipoic acid, and N-acetylcysteine indicate a beneficial impact on insulin sensitivity and offer the possibility of new treatment approaches for IR [93]. So, IR is certainly involved in the physiological process of PCOS but may well be a noninitial factor caused by OS. However, OS still remains increased in PCOS independent of obesity and IR [94, 95].

### 2.3. Oxidative Stress, Chronic Inflammation, and Polycystic Ovary Syndrome

Chronic low-grade inflammation is considered as an important feature of polycystic ovary syndrome (PCOS) and has been suggested to participate in the pathogenesis and development of PCOS [96, 97]. Inflammatory markers, such as C-reactive protein (CRP), tumor necrosis factor (TNF), interleukin-6 (IL-6), interleukin-18 (IL-18), monocyte chemotactic protein-1 (MCP-1), and acute phase serum amyloid A (APSAA), increased in women with PCOS compared with the normal [98–102]. It has been accepted that there is a tight link of oxidative stress (OS) and inflammation, and it is hard to distinguish inflammation from OS absolutely; they are usually accompanied with each other [37]. Reactive oxygen species (ROS) could induce releasing inflammatory factors and inflammatory response, via activating the associated signaling pathways of nuclear factor-*κ*B (NF-*κ*B), activated protein-1 (AP-1), and hypoxia-inducible factor-1 (HIF-1) [103]. On the other hand, ROS could be generated by rheumatoid synovial cells via the nicotinamide adenine dinucleotide phosphate (NADPH) oxidase system (Nox), during exposure to two major rheumatoid arthritis (RA) cytokines, interleukin-1*β* (IL-1*β*) and TNF-*α* [104, 105].

Inflammation has also been demonstrated to be associated with IR in PCOS [106]. It was reported that adipose-derived TNF-*α* levels in mice were increased during the advancement of obesity, but when TNF-*α* was neutralised, insulin sensitivity was improved [107]. As well as OS, inflammation could induce insulin resistance (IR) mainly via interfering with post-insulin receptor signaling pathway, insulin receptor substrate 1-phosphatidyl inositol 3 kinase-protein kinase B (IRS1-PI3K-PKB/Akt) pathway [108].

### 2.4. Oxidative Stress, Hyperandrogenemia, and Polycystic Ovary Syndrome

Hyperandrogenemia is a classical feature of polycystic ovary syndrome (PCOS), and 70%–80% of women with hyperandrogenemia are diagnosed with PCOS [109]. Hyperandrogenemia is regarded as the core pathogenesis of PCOS, as PCOS models of animals could be established by excess androgen administration [110, 111]. For the increased androgen levels in PCOS, insulin resistance (IR) is regarded as the primary factor, by compensatory hyperinsulinemia [112]. Insulin is reported to stimulate ovarian androgen secretion directly alone and/or augment luteinizing hormone- (LH-) stimulated androgen secretion [113–115]. In addition, insulin may also enhance the amplitude of gonadotropin-releasing hormone- (GnRH-) stimulated LH pulses, decrease hepatic production of serum sex hormone-binding globulin (SHBG), and/or decrease insulin-like growth factor binding protein-1 (IGFBP-1) [116–121]. Finally, the availability of free insulin-like growth factor-1 (IGF-1) is increased to stimulate androgen production [122, 123].

However, oxidative stress (OS) and inflammation seem to contribute to hyperandrogenemia in PCOS, but detailed interactions still remain unclear, as few investigations have been discovered to focus on the subject. In multi-investigations, OS and inflammation markers are discovered to be positively correlated with androgen levels in PCOS patients [124–126]. In vitro, OS was reported to enhance the activities of ovarian steroidogenesis enzymes, which could stimulate androgen generation, and antioxidative chemicals, just as statins, inhibit the activities [127]. Tumor necrosis factor-*α* (TNF-*α*), an inflammatory marker associated with tissue inflammation, was reported to have the ability to promote the proliferation of mesenchymal cells of follicular membrane and the synthesis of androgen in the rat [128].

Hyperandrogenemia seems to have the ability to cause obesity, IR, and OS in females and female animals. Compared with controls, PCOS models induced by excess androgen have increased weights, triglycerides, nonesterified fatty acid (NEFA), fasting serum insulin (FINS), fasting blood glucose (FBG), homeostasis model assessment of insulin resistance (HOMA-IR), and altered oxidative stress markers, such as malondialdehyde (MDA), glutathione (GSH), and superoxide dismutase (SOD) [129–132]. In addition, after women with normal body mass index (BMI) of reproductive age were administered with oral dehydroepiandrosterone (DHEA) to increase the androgen levels in vivo, blood samples were obtained both under fasting state and after glucose stimulation, and leukocytic reactive oxygen species (ROS) generation, p47(phox) gene expression, and plasma thiobarbituric reactive substances (TBARS) were discovered to be increased to promote oxidative stress [101]. Nuclear factor-*κ*B (NF-*κ*B) is the potential crucial mediator of inflammation induced by hyperandrogenemia [133–135]. Expression and phosphorylation level of NF-*κ*B increased, and interleukin-6 (IL-6) and monocyte chemotactic protein-1 (MCP-1) synthesis was enhanced in adipose cells after administering testosterone, but IL-6 and MCP-1 levels decreased when NF-*κ*B inhibitors were administered as well [136].

It is interesting to note that androgen may also play a role in protecting cells or tissues from inflammation and oxidative stress. In the obese PCOS patients, body mass, free fatty acid level, IL-6 level, and C-reactive protein (CRP) level increased, while androgen level was downregulated with GnRH agonist for a long term [137]. In addition, androgen was reported to have the ability to enhance the activity of hormones-sensitive lipase (HSL) to promote lipolysis and inhibit adipose tissue further growth [138]. Thus, a hypothesis was raised that androgen may contribute to anti-inflammation by promoting lipolysis, limiting adipose tissue addition, and further reducing inflammatory factor synthesis [137, 139]. In human decidual endometrial stromal cells, expressions of forkhead box protein O1 (FOXO1) and superoxide dismutase 2 (SOD2) could be promoted by dihydrotestosterone (DHT) to enhance the resistance to oxidative stress [140]. It indicates that the functions of androgen may perform multiformity in different circumstances and depend on the dosage.

## 3. Polycystic Ovary Syndrome and Cancers

A higher risk for cancers of the reproductive tract, especially endometrial cancer, seems to be related to polycystic ovary syndrome (PCOS) [141–144]. In addition, PCOS women also manifest clinical features, correlated with risk factors for breast cancer and ovarian cancer [13, 14, 145]. However, defined associations of PCOS, breast cancer, and ovarian cancer have not been found yet until recently [14]. The association of PCOS and endometrial was firstly reported in 1949, and the complicated interrelationship between endometrial cancer and PCOS has been recognized for several years, involving multiple risk factors, such as obesity, diabetes, hypertension, anovulation, nulliparity, and family history [16, 17, 146]. The meta-analysis of the data collected by Chittenden et al. [145] suggests that women with PCOS are more likely to develop cancer of the endometrium (OR 2.70, 95% CI 1.00–7.29), and the risk would increase to 3-fold, which was confirmed by Haoula et al. [143]. While the same meta-analysis was done by Fearnley et al., a similar conclusion was obtained, but the risk of endometrial cancer in PCOS women was enhanced to 4-fold (OR 4.0, 95% CI 1.7–9.3) compared with controls in another study based on Australian women younger than 50 years [147]. In addition, the increased risk for endometrial cancer in PCOS women is modified to 2.7-fold (95% confidence interval 1.0–7.3) by Amsterdam ESHRE/ASRM-Sponsored 3rd PCOS Consensus Workshop Group [148].

### 3.1. Contributions of Oxidative Stress to Cancer Pathogenesis

Oxidative stress (OS), which is altered in PCOS, increases in malignant cells compared with normal cells in culture and in vivo [149, 150]. OS could induce directly genetic variation by DNA damage, such as DNA chain rupture, base modification, DNA-DNA crosslinking, DNA-protein crosslinking, and epigenetic change, including elevated DNA methylation level, which both play important roles in the pathogenesis of cancer [12, 22]. Most modifications of DNA bases locate on the eighth carbon atom of deoxy guanine, forming 8-hydroxy-deoxyguanosine (8-OHdG). The formation of 8-OHdG could make the modified guanine replaced by thymine, leading to gene mutation and resulting in the base pairing error of “G-C→T-A” in the process of DNA replication [22, 151]. The 8-OHdG level of tumor cell is found to be significantly higher than that of normal cell and further regarded as a classical biomarker of oxidative DNA damage [152]. Though 8-OHdG could not kill cells directly, it could induce the nearby DNA bases to be modified singularly, aggravating genome instability and tumor cell transfer [153]. While adducts, just as 8-OHdG, avoid DNA self-repair by 8-oxoguanine glycosylase (OGG1) and mutY DNA glycosylase (MUTYH), genetic mutations (point mutations mainly) could be caused, and cancer would initiate if the DNA mutations locate in cancer-related genes, such as Ras protooncogene and p53 cancer suppressor gene [25, 26, 151, 154].

DNA methylation refers to the process that the methyl group of S-adenosyl-L-methionine (SAM) is transferred to adenine base or cytosine base of DNA catalyzed by DNA transmethylase (Dnmt) after DNA replication, modifying the DNA [155]. DNA methylation is involved in expression and control of genes and acts specifically according to tissue and gene. In the normal cells, the normal state of genome is held by hypomethylation levels of the promoter region of tumor suppressor genes and hypermethylation levels of some repetitive sequences, such as long interspersed nuclear element (LINE1) and Alu element [156]. DNA damage induced by reactive oxygen species (ROS), especially ^∙^OH, could influence the connection of DNA, as a substrate, with Dnmt, decreasing the methylation levels of the whole genome [26]. However, ROS also could induce hypermethylation of the promoter regions of cancer suppressor genes, promoting cell malignant transformation [157].

### 3.2. Oxidative Stress-Induced DNA Damage in Polycystic Ovary Syndrome

Micronucleus (MN) frequency, evaluated by cytokinesis block micronucleus index, which reflects genomic instability, is increased in PCOS patients compared with controls [158–161]. Furthermore, women with PCOS show a significant increase in DNA strand breakage and H_2_O_2_-induced DNA damage [162]. In addition, elevated chromosome malsegregation (assessed by X chromosome chromogenic in situ hybridisation) and reduced mitochondrial DNA (mtDNA) copy number (reflecting mitochondrial metabolism) are also found in PCOS [159, 163].

Serum MDA levels, an OS marker, were observed to be positively correlated with MN in PCOS patients but not the normal [158]. In addition, mtDNA copy number was negatively correlated with indices of insulin resistance, waist circumference, and triglyceride levels and positively correlated with sex hormone-binding globulin levels [163]. Significant correlations were also found between free testosterone and DNA strand breakage and H_2_O_2_-induced DNA damage [162]. As stated above, there are intimate interactions between OS and IR and obesity. It seems that the altered oxidative stress in PCOS has increased the instability of genes and the risk of DNA mutations and potentially contributes to the pathogenesis of gynecological cancers.

### 3.3. Obesity and Endometrial Cancer

Obesity could significantly aggravate OS and is usually accompanied with PCOS and is well known to be associated with endometrial hyperplasia and endometrial cancer, thus being regarded as one of the most significant risk factors for endometrial cancer [15]. Approximately 70–90% of Type 1 (estrogen-dependent) endometrial cancer patients are obese [164], and Schouten et al. demonstrated that obesity increased the risk of endometrial cancer by 4.5 times [165]. In fact, several studies show that adiposity contributes to the increased incidence and/or death from cancers of not only endometrium but also colon, breast, kidney, ovary, esophagus, stomach, pancreas, gallbladder, and liver [166, 167]. Furthermore, this increased endometrial cancer risk related to PCOS is reduced by almost one-half when adjusted for body mass index (BMI) (OR 2.2, 95% CI 0.9–5.7), emphasizing that obesity plays a key role in endometrial cancer pathogenesis, possibly via oxidative stress [15].

### 3.4. Insulin Resistance and Endometrial Cancer

Insulin resistance (IR), which is also significantly associated with OS regardless of obesity, is another common feature of PCOS and endometrial cancer and is regarded as the potential mechanism of endometrial hyperplasia and endometrial cancer pathogenesis in PCOS [14]. Elevated fasting serum insulin levels and insulin responses after glucose administration have been found in postmenopausal women with endometrial cancer [168]. In the study of Zhang, it is statistically significant that 12 of 19 PCOS patients with IR show endometrial hyperplasia or endometrial canceration compared to 4 of 15 PCOS patients without IR [169].

Just as stated above, IR would induce compensatory hyperinsulinemia, and excess insulin would increase insulin growth factor-1 (IGF-1). Insulin and IGF have been shown to accelerate the growth of endometrial cancer cells in vitro, and the mitogenic effect of hyperinsulinemia may be mediated by activation of the mitogen-activated protein kinase (MAPK) pathway [170], increasing expression of vascular endothelial growth factor (VEGF) [171]. Conversely, when endometrial cancer cells are exposed to serum from metformin-treated women with PCOS, cell growth is attenuated, and signaling pathways associated with inflammation and tumor invasion are altered [172]. Hyperinsulinemia reduces insulin-mediated glucose uptake and also enhances steroidogenesis. As a result, excessive insulin stimulates theca cell androgen secretion activity and elevates serum-free testosterone levels through the pathways stated above [173]. Testosterone level has been shown to be positively correlated with p-ERK and p-AKT, which are significantly higher in endometrial tissue of PCOS patients with endometrial hyperplasia or canceration compared with the normal controls, and play key roles in tumor proliferation [169]. In addition, just as discussed above, OS is an important inducer of IR by post-insulin signaling defects and has interassociation with hyperandrogenemia. Consequently, IR and hyperandrogenemia may be the potential converged mechanisms that oxidative stress influences on during endometrial canceration process.

### 3.5. Estrogen and Endometrial Cancer

The prolonged exposure to unopposed estrogen in the absence of sufficient progesterone, which is induced by denominator anovulation, is also regarded as a major factor causing endometrial hyperplasia and canceration in PCOS [174–176]. Estrogen could bind to its nuclear receptor, stimulating secretions of various growth factors, such as IGF, and epidermal growth factor (EGF), and activate ERK signaling pathway, to promote endometrial proliferation and even canceration. In addition, metabolites of estrogen also could be the inducers of endometrial canceration by binding to DNA and causing further DNA damage, and the procedure is associated with oxidative stress. Under oxidative stress, estrogen intermediate metabolites, including 2-hydroxyl estrone (2-OHE1), 4-hydroxyl estrone (4-OHE1), and 16*α*-hydroxyl estrone (16*α*OHE1), could not be methylated and eliminated from the body and would be oxidized to semiquinonoid compounds and quinonoid compounds. The two abnormal types of metabolites of estrogen with electron affinity bind to nucleophilic group of DNA by covalent bond, causing DNA mutation, and further lead to endometrial canceration process.

### 3.6. Polycystic Ovary Syndrome and Other Cancers

In the investigation of Schildkraut et al. [142], ovarian cancer risk is found to increase to 2.5-fold (95% confidence interval [CI] 1.1–5.9) among women with PCOS, and the association is found to be stronger among women who never used oral contraceptives (odds ratio [OR] 10.5, 95% CI 2.5–44.2) and women who were in the first quartile of body mass index (13.3–18.5 kg/m^2^) at the age of 18 (OR 15.6, 95% CI 3.4–71.0). Though PCOS perhaps could increase the risk of ovarian cancer based on the limited few studies, the association of them has also been under the doubt and needs more evidences to be proved. On the other hand, breast cancer seems to be not associated with PCOS based on the current limited data [14]. In addition, powerful evidences are needed to evaluate the associations between PCOS and vaginal, vulvar, and cervical cancer or uterine leiomyosarcoma. Nevertheless, obesity and estrogen excess are suggested as the two important factors inducing cancers besides endometrial cancer [14].

## 4. Conclusion

**Figure 1:**
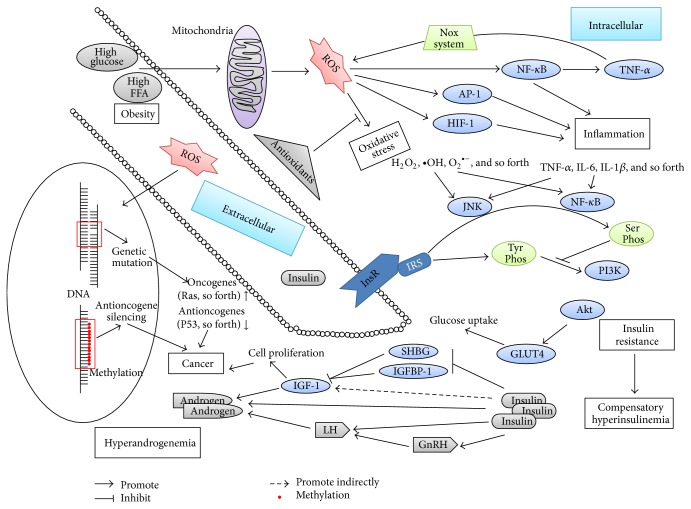
Interactions of oxidative stress, inflammation, insulin resistance, and hyperandrogenemia are described briefly in the figure, which are all involved in polycystic ovary syndrome physiopathology. Oxidative stress seems to induce cancer through genetic variation and cell signaling pathway. FFA, free fatty acid; ROS, reactive oxygen species; NF-*κ*B, nuclear factor kappa B; AP-1, activator protein-1; HIF-1, hypoxia-induced factor-1; TNF-*α*, tumor necrosis factor-*α*; Nox, nicotinamide adenine dinucleotide phosphate oxidase system; IL, interleukin; JNK, c-Jun N-terminal kinase; InsR, insulin receptor; IRS, insulin receptor substrate; Tyr Phos, tyrosine phosphorylation; Ser Phos, serine phosphorylation; PI3K, phosphatidyl inositol 3-kinase; Akt, protein kinase B; GLUT4, glucose transporter-4; GnRH, gonadotropin-releasing hormone; LH, luteinizing hormone; SHBG, sex hormone-binding globulin; IGFBP-1, insulin growth factor binding protein; IGF-1, insulin growth factor-1.

It is known that DNA damage and methylation induced by oxidative stress (OS) play key roles in the early stage of tumor pathogenesis and tumor conversion by activating protooncogene and silencing antioncogene. Mechanistically, the abnormal oxidative stress in polycystic ovary syndrome (PCOS) patients could cause genetic instability and raise the risk of cancers. OS has been demonstrated to be significantly associated with obesity, insulin resistance (IR), inflammation, and hyperandrogenemia, which are the common characteristics and potential inducers of PCOS and endometrial cancer and could participate and be induced in an interweaving way during disease physiology (Figure 1). ROS and proinflammatory factors, produced under OS, could induce IR majorly through IRS-PI3K-Akt by activation of associated signaling pathways, such as NF-*κ*B and JNK. Hyperinsulinemia, compensatory for IR, contributes to cancer pathogenesis by activating cell proliferation signaling pathways and finally leads to malignant transformation. In addition, OS, IR, and inflammation could be induced by excess androgen in vivo and involved in obesity. Thus, OS is considered as an initial factor, leading to cancers in PCOS. It remains to be determined whether other potential pathways mediated by oxidative stress could play roles in the pathogenesis of PCOS related cancers.

## Figures and Tables

**Table 1:** Oxidative stress (OS) markers employed in polycystic ovary syndrome (PCOS) patients are shown in the table.

Biomarkers evaluating OS level	Location and source	OS levels of PCOS patients compared with the normal		References
			Independent of obesity	
*Markers reflecting oxidative levels*				
Malondialdehyde (MDA)	Serum; erythrocyte	Higher	Higher	[4, 42, 43, 69, 126, 158, 177–182]
Advanced glycosylated end products (AGEs)	Serum	Higher		[177, 183]
Xanthine oxidase (XO)	Serum	Higher		[184]
8-Hydroxydeoxyguanosine (8-OHdG)	Serum	Lower	Lower	[185]
Lipid peroxidation (LPO)	Follicular fluid; serum	Higher		[178, 186]
Protein carbonyl	Serum	Higher		[187]
Reactive oxygen species (ROS)	Follicular fluid; granulose cell; mononuclear cell	Higher		[186, 188, 189]
Total oxidant status (TOS)	Serum	Higher	Higher	[190, 191]
Oxidative stress index (OSI)	Serum	Higher		[190]
Homocysteine (Hcy)	Serum	Higher	Higher	[4]
Asymmetric dimethylarginine (ADMA)	Serum	Higher	Higher	[4]
Prolidase (PLD)	Serum	Higher		[190]
Nitrotyrosine (Ntyr)	Serum		Higher	[192]
Uric acid	Serum		Higher	[192]
Neopterin (NEO)	Serum	Higher	Higher	[193]

*Markers reflecting antioxidative levels*				
Superoxide dismutase (SOD)	Serum; erythrocyte; follicular fluid	Higher	Higher	[4, 42–44, 182, 194, 194]
Glutathione (GSH)	Serum	Lower	Lower	[4, 43]
Paraoxonase 1 (PON1)	Serum	Lower	Lower	[4, 69, 179, 184]
Heme oxygenase-1 (HO-1)	Serum		Lower	[195]
Total antioxidant status (TAS)	Serum	Lower	Lower	[126, 187]
Total antioxidant capacity (TAC)	Follicular fluid; serum	Lower		[69, 186]
Vitamin E	Serum	Lower		[178]
Vitamin C	Serum	Lower		[178]
Thiol	Serum	NS	Lower	[94, 184]
L-Carnitine	Serum		Lower	[196]
